# Draft Genome Sequences of Six Vibrio diazotrophicus Strains Isolated from Deep Subsurface Sediments of the Baltic Sea

**DOI:** 10.1128/genomeA.00081-18

**Published:** 2018-03-08

**Authors:** Daniel Castillo, Verona Vandieken, Bert Engelen, Tim Engelhardt, Mathias Middelboe

**Affiliations:** a Marine Biological Section, University of Copenhagen, Helsingør, Denmark; b Institute for Chemistry and Biology of the Marine Environment (ICBM), University of Oldenburg, Oldenburg, Germany

## Abstract

We present here the draft genome sequences of six Vibrio diazotrophicus strains, which were isolated from deep subseafloor sediments of the Baltic Sea. The genomic sequences contained several virulence and antibiotic resistance genes. These genome sequences provide insights into the genetic composition and evolution of the genus Vibrio in marine environments.

## GENOME ANNOUNCEMENT

Marine subsurface sediments cover approximately 70% of the Earth’s surface and harbor a remarkable population of microbial life that comprises 1/10 of all living biota on Earth (1). Studies of adaptation have suggested that the microbes found in the subsurface seabed represent descendants of surface communities that were buried in the past (2). Thus, subseafloor sediment provides an unprecedented opportunity to investigate genetic richness of bacterial communities on a time scale of thousands to millions of years.

Vibrio species are heterotrophic bacteria that occur naturally in diverse marine environments, including subseafloor sediments (3). Although the presence of Vibrio spp. has been determined by 16S rRNA gene library analysis in deep marine subsurface sediments (4), little is known about the whole-genome sequences of Vibrio spp. isolated from this environment. Here, we announce the draft genome sequences of six Vibrio diazotrophicus strains isolated from deep subsurface sediments (12.7 to 79.6 m below the sea floor) of the Baltic Sea.

Vibrio diazotrophicus strains 60.6B, 60.6F, 60.18M, 60.27F, 65.7M, and 65.10M were grown at 22°C in LB broth (catalog no. 12106 to 12105; Mo Bio, Inc.). Genomic DNA was extracted by using the Wizard Genomic DNA purification kit (catalog no. A1120; Promega) according to the manufacturer’s protocol. A sequencing library was prepared using the Illumina HiSeq 2500 platform (BGI, Hong Kong) with paired-end read sizes of 100 bp. A total of 15,092,729 to 17,078,579 paired-end reads were used for *de novo* assembly in Geneious version 9.1.6 (5). Short and low-coverage contigs were filtered out, resulting in a set from 37 to 77, with an average coverage of 99× (average *N*_50_ value, 137,585 bp). Annotation was performed with the NCBI Prokaryotic Genome Annotation Pipeline (PGAP) (6). Additionally, the genomes were analyzed on the Rapid Annotations using Subsystems Technology (RAST) server (7). Antibiotic resistance genes were identified using ResFinder version 2.1 (8), virulence factors were identified using VirulenceFinder version 1.2 (9), and prophage-related sequences were identified using PHASTER (10).

The final assemblies for the six Vibrio diazotrophicus strains had total lengths from 4,543,538 to 4,824,458 bp and an average G+C content of 43.4%. Genome annotations resulted in between 4,122 to 4,421 coding sequences (CDSs), 56 to 67 tRNAs, and 3 to 5 rRNAs for all strains. Three *zot*-encoding prophages of 6.9, 9.1, and 12.5 kb were detected for strains 60.6B, 60.27F, and 60.18M, respectively. Interestingly, strain 60.6B contained the virulence-related genes *toxR* and *toxS*, which were described previously in V. cholerae (11). Genes associated with resistance to the antibiotic fluoroquinolone (*parC, parE*, *gyrA*, and *gyrB*) were found for all strains except 60.27M. Tetracycline resistance genes (*BLc* and *BLII*) were only found in strains 60.27F and 65.10M. Also, all strains harbored the eight genes (*R1*, *DedA*, *R3*, *R4*, *R5*, *DedD*, *R8,* and *PurF*) related to colicin V production. Thus, these genome sequences can facilitate understanding of the genetic diversity within the genus Vibrio in one of the largest ecosystems on Earth, the deep marine biosphere.

### Accession number(s).

Genomes were deposited in the DDBJ/EMBL/GenBank databases under the accession numbers listed in Table 1. The assembly versions described in this paper are the first versions of the assemblies.

**TABLE 1:** Summary report of the *de novo* assembly of the six Vibrio diazotrophicus strains from this study

Strain	No. of contigs	*N*_50_ value	Assembly length (bp)	G+C content (%)	Coverage (×)	No. of CDSs^a^	No. of tRNAs	Accession no.
65.7M	54	196,201	4,606,601	43.4	98	4,159	66	POSL00000000
65.10M	77	114,453	4,824,458	43.5	91	4,421	67	POSM00000000
60.27F	42	254,877	4,691,444	43.5	107	4,267	67	POSK00000000
60.18M	40	193,656	4,544,576	43.3	100	4,085	56	POSJ00000000
60.6F	49	180,451	4,747,742	43.5	98	4,300	66	POSI00000000
60.6B	37	206,116	4,543,538	43.7	110	4,122	65	POSH00000000

a CDSs, coding sequences.
